# Validation of the nurse leadership and organizational culture (N-LOC) questionnaire

**DOI:** 10.1186/s12913-019-4290-z

**Published:** 2019-07-09

**Authors:** Juliana Nga Man Lui, Janice Mary Johnston

**Affiliations:** School of Public Health, Li Ka Shing Faculty of Medicine, The University of Hong Kong, Hong Kong SAR, China

**Keywords:** Leadership style, Organizational culture, Competing values framework, Multifactor leadership theory, Nurses

## Abstract

**Background:**

Leadership style and organizational culture have often been studied independently in nursing research despite abundant evidence that the two factors both influence employee outcomes. Moreover, diverse theoretical typology and measuring instruments challenges generalizability of findings. Employees from different cultural, geographical, occupational settings were also reported to have varying interpretation on organizational culture and leadership style underlying constructs. This study aims to validate the Nursing Leadership and Organizational Culture (N-LOC) questionnaire, based on the two commonly used theoretical frameworks: Multifactor Leadership Theory and Competing Values Framework, on its applicability in an Asian hospital setting.

**Methods:**

All full-time nurses from two distinctive Asian hospitals (H1: *n* = 295 and H2: *n* = 1146) were invited to participate in this questionnaire study. Exploratory factor analysis (EFA) was carried out when confirmatory factor analysis (CFA) fit indices were not satisfactory after model refinement to explore the actual underlying construct in sampled population. Part-time and outsourced nurses were excluded. 93 nurses from H1 were randomly selected for test-retest reliability 4 weeks post initial survey. Scale internal consistency, convergent and discriminant validity were also assessed.

**Results:**

CFA results indicated that the proposed CVF organizational culture 4-factor structure was applicable to our sample but not the MLQ leadership 3-factor/9-factor structure. EFA revealed a 2-factor leadership style construct for our sample, named Confucius transformational and Laissez-Faire passive leadership. Transformational leadership traits already embedded in Confucius cultural values (self-sacrifice, stresses collective mission, instills pride) did not apply, the new Confucius transformational construct which resembles LMX theory paternalistic leadership style is deemed more suitable in an Asian context. A final 14-item 2-factor leadership and 13-item 4-factor organizational culture construct was yielded with satisfactory fit indices (CFI, TLI > 0.95, RMSEA < 0.08), internal consistency (Cronbach’s alpha > 0.7), test-retest reliability (ICC > 0.4) and convergent and discriminant validity.

**Conclusion:**

A reliable N-LOC organizational culture and leadership questionnaire (N-LOC) has been validated in an Asian nurse context. Study results demonstrated the importance of scale validation in cross-cultural adaptation, as underlying scale constructs may change with specific cultural and contextual factors. Future studies are encouraged to test the adaptation of this scale in other cultural and occupational settings.

**Electronic supplementary material:**

The online version of this article (10.1186/s12913-019-4290-z) contains supplementary material, which is available to authorized users.

## Background

Leadership style is defined as the interactive influence of accepted leaders on employees to achieve desired organizational outcomes [1], while organizational culture is a reflection of a set of shared fundamental beliefs, assumptions and common practices [2]. Despite abundant evidence in literature that leadership and organizational culture are two interlinked factors influencing employee outcomes (employee effectiveness, commitment, performance and satisfaction) in organizational theory [3–6], these two factors were often studied independently in nursing research [7–9].

Diverse theoretical typology and measuring instruments for leadership style and organizational culture constructs challenges cross-study comparisons and generalizability of findings [8, 10–17]. Moreover, employees from diverse geographical, occupational settings and cultural values were reported to have varying interpretation on organizational culture types and leadership style underlying constructs [18–21]. Existing organizational culture and leadership style theories and questionnaires are well-established in Western business settings [22, 23], however the generalizability and validity of these constructs remain underexplored in an Asian hospital setting.

Multifactor Leadership Theory (MLT), a commonly used leadership theory in nursing research, classifies leadership styles into three categories: transformational, transactional, and Laissez-Faire. Transactional leaders guide their employees to work within existing organizational cultures by providing performance-based reward. Transformational leaders alter existing organizational cultures to align with a new inspirational vision, motivating followers to demonstrate self-conscious performance [24]. Lassez-Faire leaders avoid taking up leadership responsibilities, or provides minimal guidance, lack of oversight, being absent when subordinates are in need of leadership and only responsive to major problems [15]. Transformational leadership is the most effective leadership style relating with positive employee outcomes such as improved work productivity, while having a more long-lasting effect on organizational outcomes than transactional leadership style [24]. Whereas Laissez-Faire leadership has been described as the least effective style, relating with lowered employee productivity and work engagement [25].

The Competing Values Framework (CVF) is a commonly used organizational culture theory used in healthcare research [7, 8]. CVF categorizes organizational culture into four distinct typologies: group, developmental, rational and hierarchical [23]. Group culture focuses on internal employee concerns and encourages commitment through teamwork, participation, and communication [26]. Organizations with group cultures are employee-centric and provide a friendly and warm family-like environments. Organizations with rational cultures are results-oriented, encouraging a competitive and effective atmosphere to aim at gaining market share. Organizations with developmental cultures focus on innovation and entrepreneurship, encouraging constant changes and risk-taking behaviour throughout the company. Organizations with hierarchical cultures are rules-oriented, promoting standardized timely procedural outputs guided by internal procedures and policies. Different organizational culture types may yield varying employee outcomes, nurses working in group-oriented cultures were found to have higher work engagement and lower turnover intention than those working in hierarchical dominant cultures [7, 8]. The CVF does not favor one culture type and suggests the distinct organizational cultures may exist concurrently within the institution [23].

This study aims to validate the Nursing Leadership and Organizational Culture (N-LOC) questionnaire, based on the two commonly used theoretical frameworks: MLT and CVF, on its applicability in an Asian hospital setting.

## Methods

### Setting

Two acute care hospitals in Hong Kong with distinctive organizational structures, hospital size, patient characteristics, management style and workplace demands were selected for this validation study. The historical and contextual characteristics of the two hospitals are similar - each having a long operating history (> 100 years), similar religious affiliation, mission and vision statements.

### Sample

The pilot study was carried out among all full-time nurses at H1 (*n* = 295) for model fitness testing and refinement purposes. It is recommended to have a sample size of ten cases per item for CFA analysis [27], and to confirm the construct validity of the reduced questionnaire in a separate and distinct study population [28, 29], therefore data collected from all full-time nurses from H2 (*n* = 1146) in the main study was used to confirm the generalizability of the reduced questionnaire structure from the pilot study at H1. Part-time and outsourced nurses were excluded in this study.

Survey packets containing: 1) an information sheet, 2) a unique identification number (UIN) labelled survey, 3) a self-sealed return envelope, and 4) a response incentive (valued at less than 1USD each) were prepared and delivered to each nurse’s primary work location. A UIN was generated for each nurse based on nurse roster information prepared by the central nursing department at each hospital, only the senior research assistant had access to the master data file linking the UIN to the personal data necessary to prepare UIN labelled surveys, return envelopes, and follow up surveys for non-responders. All data used for analysis was de-identified. Follow up questionnaires were sent to non-respondents one-month post-initial contact to achieve highest possible response rates.

### Nurse leadership and organizational culture (N-LOC) questionnaire

The preliminary questionnaire included 62 items (leadership style: 36 items, organizational culture: 20 items, demographics: 6 items). Leadership style was measured by MLQ rated on a 7-point Likert scale (1, Not at all - 7, frequently, if not always) [22]. The previously validated MLQ has three domains:- transformational, transactional and Laissez-Faire, and nine subdomains:- contingent reward (CR) (4 items), management by exception active (MBEA) (4 items), management by exception passive (MBEP) (4 items), individualized consideration (IC) (4 items), idealized influence attributed (IIa) (4 items), idealized influence behavior (IIb) (4 items), inspirational motivation (IM) (4 items), intellectual stimulation (IS) (4 items) (subdomain descriptions listed in Additional file 1: Table S1). Item wordings were not shown due to MLQ questionnaire copyright restrictions (license was purchased for questionnaire copies in this study).

Organizational culture was measured by CVF questionnaire rated on a 5-point Likert scale (1:strongly disagree – 5: strongly agree) [8], and includes four domains: group (5 items), developmental (5 items), rational (5 items) and hierarchical (5 items) [23] (domain descriptions listed in Additional file 1: Table S1). Demographics items asked for nurses’ age, gender, shift, education, rank and primary working location.

### Translation and content validity

The questionnaire was back-translated from English - Cantonese Chinese - English by two public health researchers. The translation and content validity were assessed by a panel of seven international and local experts, including Cantonese-speaking nurses, nursing managers and psychometrics experts, and moderated by an ‘editor-in-chief’ to achieve consensus.

### Model fitness and refinement

Confirmatory factor analysis (CFA) using maximum likelihood estimation and pairwise deletion of missing values was used to establish scale factor structures [30]. To improve model fitness, items with factor loading < 0.4 and standardized residual covariance > 1.96 or < − 1.96 (*p* < 0.05) were deleted. Between error variance paths were added if modification indices were greater than six and supported by theory or prior research [31].

#### CFA fit indices

Model fit was evaluated using the comparative fit index (CFI), Tucker Lewis index (TLI), Goodness-of-fit index (GFI), standardized root-mean-square residual (SRMR) and root mean square error of approximation (RMSEA). Test values of 1) CFI, TLI and GFI > 0.90 and 2) SRMR and RMSEA < 0.08 were used to assess model fit [32].

For scales that did not yield satisfactory CFA fit indices after the model refinement process an exploratory factor analysis (EFA) with Oblimin rotation was used to identify the underlying construct. Items with low factor loading (< 0.4) and items that cross-loaded across multiple domains were removed.

### Test-retest reliability

Ninety-three nurses were randomly selected in H1 to carry out test-retest reliability using intra-class correlation coefficient (ICC) two-way random measurement. Sample size for test-retest reliability was determined by the “pwr” R software package, assuming alpha of 0.05, power of 0.8 and dropout rate of 60%. Moderate and excellent reliability were indicated by ICC values of 0.4–0.74, and ≥ 0.75 respectively. Internal consistency of the domains and subdomains was evaluated using Cronbach’s alpha coefficient with > 0.7 considered acceptable and over 0.8 excellent.

### Convergent and discriminant validity

Convergent validity was supported if 1) group, rational and developmental culture were positively associated with transformational leadership, and 2) hierarchical leadership was positively correlated with Laissez-Faire leadership. While discriminant validity was supported if 1) group, rational and developmental cultures were negatively correlated with Laissez-Faire leadership, and 2) Lasseiz-Faire was negatively correlated with transformational leadership.

### Statistical analysis

CFA was performed in R (version 3.4.1) using the “lavaan” package (version 0.5–23.1097). EFA, Cronbach alpha, ICC values and Pearson correlation coefficients were calculated using SPSS version 24.

### Ethics approval

Ethics approval was obtained from the Institutional Review Board of the University of Hong Kong/Hospital Authority Hong Kong West Cluster (HKU/HA HKW IRB) (reference number: UW 16–102) and Hospital Authority Kowloon West Cluster Research Ethics Committee (reference number: KW/EX-17-028(108–07)).

## Results

One-hundred and eighty-five of 295 nurses at H1 (RR_H1_ = 62.7%) completed the preliminary 62-item N-LOC questionnaire and 824 of 1146 nurses at H2 (RR_H2_ = 71.9%) completed the reduced 33-item N-LOC questionnaire (after model fitness and refinement process, results presented below). About 90% of nurses were female in both hospitals, whereas H2 vs H1 nurses were significantly younger, in more senior staff positions and more highly educated (Table 1). Seventy-one of 93 nurses at H1 (RR = 76.3%) completed the test-retest questionnaire.

**Table 1:** Nurse demographics and work characteristics at H1 and H2

Characteristics	H0 (*n* = 185) n(%)	H1 (*n* = 824) n(%)	*P*-value
Gender			
Male	19 (10.3)	95 (11.5)	0.72
Female	166 (89.7)	729 (88.5)	
Age Group			
≤ 21–30	20 (16.3)	226 (28.4)	< 0.001
31–40	38 (30.8)	196 (24.6)	
41–50	28 (22.8)	263 (33.0)	
51- ≥60	37 (30.1)	111 (13.9)	
Education			
Certificate/ Diploma	39 (28.3)	72 (9.1)	< 0.001
Associate Diploma/ Higher Diploma	27 (19.5)	47 (6.0)	
Bachelor’s Degree (BscN/BN)	48 (34.8)	432 (54.8)	
Postgraduate Degree	24 (17.4)	237 (30.1)	
Nurse Ranking			
Junior staff (EN)	58 (31.4)	51 (6.2)	< 0.001
Junior staff (RN)	98 (53.0)	566 (68.7)	
Middle management (APN/NC)	16 (8.6)	172 (20.9)	
Senior management (WM/UM/DOM)	13 (7.0)	35 (4.2)	
Working Schedule			0.08
Shift schedule	116 (76.3)	664 (82.8)	
Regular schedule (9 am-6 pm)	36 (23.7)	138 (17.2)	
Department			< 0.001
A&E^a^	–	46 (5.9)	
GOPC/SOPC	14 (7.5)	48 (6.1)	
Medicine^b^	84 (45.4)	309 (39.6)	
Surgery^c^	68 (36.8)	303 (38.8)	
Others^d^	19 (10.3)	75 (9.6)	

^a^ *A&E* Accident and emergency

^b^ Medicine – includes medicine, geriatrics, pediatrics and intensive care unit (ICU)

^c^ Surgery – includes surgery, obstetrics, gynecology and operation theatre

^d^ 0thers – includes administration, management, residential care, public health, rehabilitation, occupational Health, community nursing, mental health, psychiatry, addiction treatment and others

### Construct validity and internal consistency

#### Leadership scale

CFA of the preliminary 36-item leadership scale for H1 was not satisfactory for the 9-factor subdomain structure as the five distinct transformational subdomain structures fail to form, cross-loading with each other. Two items belonging to the transactional MBEP subdomain asking on absence in leadership until problem arises or becomes persistent, loaded on the Laissez-Faire domain. All items belonging to the transactional MBEA subdomain loaded across multiple factors. and two transactional CR subdomain items asking on whether leaders spend time mentoring and considers individual capability differences cross-loaded with the transformational subdomains. Similarly, the 3-factor domain structure previously proposed by Kanste (Table 2) [27, 32] did not converge to a satisfactory domain structure.

**Table 2:** Confirmatory factor analysis results of leadership and organizational culture scales

Factors	Hospital	CFI	TLI	GFI	RMSEA	SRMR
Leadership Scale						
Before retesting with EFA						
9 factor (Bass)	H1	0.773	0.744	0.918	0.091 (0.085–0.097)	0.121
3 factor	H1	0.758	0.742	0.915	0.092 (0.086–0.097)	0.099
3 factor reduced ^a^	H1	0.779	0.759	0.948	0.100 (0.092–0.107)	0.099
After retesting with EFA						
2 factor	H1	0.907	0.895	0.954	0.076 (0.066–0.086)	0.056
	H2	0.902	0.890	0.929	0.101 (0.097–0.105)	0.039
Organizational Culture						
4 factor (5 items)	H1	0.812	0.782	0.978	0.106 (0.096–0.117)	0.079
4 factor (after reduction)	H1	0.969	0.959	0.995	0.054 (0.030–0.075)	0.046
	H2	0.948	0.931	0.994	0.073 (0.066–0.082)	0.045

^a^ after deleting item loadings < 0.4 and standardized residuals > 1.96 or < −1.96

Thus, EFA was carried out on H1 data to explore the possibility of a different underlying construct in our population. However, multiple cross-loading items occluded the underlying factor structure, therefore model refinement procedures were carried out (deleting items with factor loadings < 0.40 followed by cross-loading items), yielding a final 14-item two-factor structure, which accounts for 64 and 71.8% of the total variance at H1 and H2 respectively (Table 3). The two factors were named ‘Laissez-Faire passive leadership’ (6 items: 2 MBEP and 4 LF items) and ‘Confucius transformational leadership’ (8 items: 1IIa, 1 IIb, 2 IS, 2 IC and 2 IM items). Mean scores and standard deviations of items and domain constructs are listed in Table 4. The original transformational leadership items that convey collective sense of group belonging or self-sacrificing spirit (e.g. IIa items where supervisors “extends beyond self-interest”, “instills pride”, IIb items where supervisors “stresses on collective mission”, “have strong purpose”) did not load onto the new Confucius-transformational domain.

**Table 3:** Exploratory factor analysis results (oblimin rotation) for leadership scale at H1 and H2

Leadership Style ^a^	H1	H2
	(64.0% variance)	(71.8% variance)
Confucius Transformational		
Promotes development	0.865	0.867
Encourages different problem approaches	0.855	0.820
Considers individual differences	0.851	0.856
Brings up new ways to finish tasks	0.837	0.853
Articulate vision	0.795	0.900
Considers morality of decisions	0.780	0.756
Behaviour earns our respect	0.690	0.687
Confident in reaching goals	0.708	0.879
Laissez- Faire-passive		
Reacts only to persistent problems	0.817	0.811
Reacts when problems arise	0.797	0.831
Avoids deciding	0.774	0.928
Unavailable when needed	0.756	0.853
Stay away from issues	0.756	0.837
Delays responding	0.712	0.876

^a^ Brief item labels were only provided for leadership scale due to copyright restrictions

**Table 4:** Means, standard deviations of items and domains

	H1	H2
Leadership Style ^a^	Mean (sd)	Mean (sd)
Lasseiz Faire Passive (LFP)	**1.96 (0.83)**	**1.96 (0.99)**
Reacts when problems arise	1.96 (1.13)	1.91 (1.21)
Reacts only to persistent problems	2.28 (0.99)	2.24 (1.11)
Stay away from issues	2.02 (0.96)	1.93 (1.09)
Unavailable when needed	1.99 (1.14)	2.01 (1.13)
Avoids deciding	1.76 (1.10)	1.89 (1.14)
Delays responding	1.76 (1.08)	1.81 (1.16)
Confucius transformational (CT)	**2.12 (0.73)**	**1.87 (0.80)**
Behaviour earns our respect	2.09 (0.99)	1.85 (0.99)
Considers morality of decisions	2.22 (0.89)	2.04 (0.90)
Encourages different problem approaches	2.14 (0.90)	2.00 (0.93)
Brings up new ways to finish tasks	2.02 (0.89)	1.93 (0.93)
Considers individual differences	2.06 (0.96)	1.88 (0.97)
Promotes development	2.01 (0.96)	1.81 (0.99)
Articulate vision	2.07 (0.85)	1.62 (0.99)
Confident in reaching goals	2.40 (0.80)	1.85 (0.97)
Organizational Culture		
Group (GRP)	**3.09 (0.64)**	**2.84 (0.69)**
Our hospital deals with the changes on the work-related procedure and process flexibly	3.12 (0.81)	2.60 (0.92)
The relationship among all staffs is built on the basis of strong mutual confidence and cooperation	2.95 (0.90)	2.89 (0.91)
When performing a task, departments in the hospital cooperate with one another well	3.04 (0.81)	2.79 (0.87)
Our nurses has confidence in other nurses’ job ability	3.23 (0.86)	3.06 (0.81)
Rational (RAT)	**3.21 (0.69)**	**2.90 (0.73)**
Our hospital is well organized to facilitate functions of each specialty	3.03 (0.77)	2.81 (0.84)
Our hospital continuously pursues new methods in order to perform works effectively	3.09 (0.83)	2.70 (0.89)
Our hospital has the detailed manual of the work	3.50 (0.81)	3.20 (0.84)
Developmental (DEV)	**2.79 (0.70)**	**2.67 (0.77)**
On behalf of the development of the hospital, our staff is willing to give up their own interest	2.62 (0.94)	2.54 (0.90)
Our staff shares the goal of the hospital and struggles to accomplish it	2.90 (0.77)	2.71 (0.85)
Our staff has strong self-confidence on the hospital image	2.84 (0.79)	2.75 (0.89)
Hierarchical (HIE)	**3.62 (0.73)**	**3.60 (0.77)**
A bureaucratic mood still exists in our hospital	3.68 (0.88)	3.70 (0.97)
It takes a relatively long time for the experienced staff from outside to adjust to the culture of our hospital	3.60 (0.84)	3.43 (0.88)
It often takes long time for top-level managers to reflect lower level staff’s opinions in decision making	3.59 (0.94)	3.65 (0.98)

^a^ Brief item labels were only provided for leadership scale due to copyright restrictions

The boldface represents the composite average score for the relevant domain

The new two-factor structure was subsequently tested in H2 and satisfactory fit indices were obtained: CFI, TLI and GFI > 0.90, RMSEA and SRMR < 0.08 (except for H2 RMSEA =0.10). Cronbach alpha for Laissez-Faire Passive was 0.92 for H1 and 0.94 for H2 and for Confucius transformational 0.87 for H1 and 0.93 for H2.

#### Organizational culture scale

CFA on H1 data yielded a 13-item 4-factor structure after model refinement procedures: group culture (4 items), rational culture (3 items), development culture (3 items), and hierarchical culture (3 items) (Table 2). The 13-item 4-factor structure organizational culture scale was also tested using CFA with H2 data and yielded satisfactory fit indices.

Cronbach alpha for each domain by hospital (H1 and H2) were as follows: for group culture 0.76 and 0.80, for rational culture 0.82 and 0.81, for developmental culture 0.79 and 0.85, and for hierarchical culture 0.75 and 0.75.

### Test-retest reliability

Four-week test-retest ICC domain scores for the leadership and culture scales at H1 achieved moderate reliability, ranging between 0.51–0.71.

### Convergent and discriminant validity

Convergent validity was supported, where group, rational and developmental cultures were moderately positively associated with each other (Table 5). Whereas weak to moderate positive correlations were observed between 1) hierarchical culture and Laissez-Faire passive leadership and 2) group, rational and developmental culture with Confucius-Transformational leadership.

**Table 5:** Correlation matrix of leadership style and organizational culture factor constructs

H1	TF	LFP	GRP	RAT	DEV	HIE
Leadership Style						
Confucius Transformational (CT)	1					
Lasseiz-Faire Passive (LFP)	−.433^a^	1				
Organizational Culture						
Group (GRP)	.435^a^	−.331^a^	1			
Rational (RAT)	.431^a^	−.327^a^	.612^a^	1		
Developmental (DEV)	.454^a^	−.206^a^	.669^a^	.584^a^	1	
Hierarchical (HIE)	−.398^a^	.369^a^	−.315^a^	−.261^a^	−.296^a^	1
H2	TF	LFP	GRP	RAT	DEV	HIE
Leadership Style						
Confucius Transformational (CT)	1					
Lasseiz-Faire Passive (LFP)	−.555^a^	1				
Organizational Culture						
Group (GRP)	.459^a^	−.392^a^	1			
Rational (RAT)	.458^a^	−.383^a^	.708^a^	1		
Developmental (DEV)	.371^a^	−.272^a^	.624^a^	.585^a^	1	
Hierarchical (HIE)	−.211^a^	.297^a^	−.234^a^	−.196^a^	−.167^a^	1

^a^Correlation is significant at the 0.01 level (2-tailed)

Divergent validity was also demonstrated, Confucius transformational and Laissez-Faire passive subdomains within the leadership style domain showed moderate to strong negative correlation (H1: -0.433, H2: − 0.555) with each other. Whereas group, rational and developmental cultures showed weak to moderate negative correlations with hierarchical culture. Weak to moderate negative correlations were also observed between the scales for 1) hierarchical culture and transformational leadership and 2) group, rational and developmental culture with Laissez-Faire passive leadership.

## Discussion

A reliable leadership and organizational culture questionnaire (N-LOC) was developed and validated amongst nurses working in two independent public hospitals in Asia. High internal consistency was achieved. Convergent and divergent validity was supported within and between scales. Several cross-cultural and occupational scale adaptation challenges occurred during the leadership scale validation process.

### Leadership style scale factor structure

The new 2-factor leadership domain structure extends Luo’s findings in a Chinese hotel industry leadership scale validation study, in which the structure applies across occupations in an Asian setting [21]. This confirms the importance of testing questionnaire validity and reliability when applying adopted questionnaires in different cultural or occupational contexts.

The new Confucius transformational leadership domain includes elements within the paternalistic leadership style under the Leader-Member Exchange theory (LMX) [33], studies showed resemblance between transformational and paternalistic leadership styles [34]. Paternalistic leadership is strongly rooted in Confucian principles fundamental in Asian cultures, such as “filial piety” (respect to parents, seniors and ancestors), “humaneness” (care to others) and “ritual consciousness” (respect for rituals in regulating righteous human actions and morale (li)). Despite shared elements with transformational leadership such as 1) challenging subordinates intellectually, 2) taking care of subordinate emotions and 3) communicating vision, other transformational leadership elements such as delegation and empowerment do not apply in both paternalistic leadership and our new Confucius transformational leadership domain [35].

Chen observed that communicating vision across organizations may not be a critical component in paternalistic leadership in Asian settings, as employees already seek common cultural value goals based on Confucian principles [35]. This may also explain why certain MLQ items (e.g. IIb item “stresses collective mission”, IIa item “instills pride as their subordinates”) failed to load on the Confucius transformational leadership domain, as such virtues are already embedded in Asian culture and not deliberately emphasized by leaders.

Furthermore, other distinctive features of paternalistic leadership include centralized decision-making, high-power distance and non-questioning subordination amongst employees [33]. Leaders pose as authoritative figures in Asian culture [35], thus items (e.g. IIa item” builds respect”, IM items “articulates future vision” and “expressing confidence in goal attainment”) using terminology such as “articulate” or “expressing confidence” fits into the Confucius transformational construct, but IM items using terminology such as “talks optimistically/enthusiastically” do not. Items written in colloquial phrases such as MBEP item “ ain’t broke, don’t fix “ may not readily apply in other cultural contexts and did not load into our finalized constructs. This highlights the importance of translation and cultural understanding when using scales developed for the West in the East.

Research on paternalistic leadership effectiveness applies to both Eastern and Western countries (China, Turkey, Mexico, Pakistan, Russia, Romania, United States, Germany, Israel and Canada) [36–39]. Nonetheless, availability of valid and standardized scales on paternalistic leadership are limited. Items from existing paternalistic leadership scales were found to resemble with MLQ items [33]. Several MLQ factor structure challenges are universal despite demographic differences in sampled populations. Transactional MBEA and CR subdomains resulted in high cross-domain correlations, contributing to construct instability and thus low scale reliability [40–42]. These two domains did not remain after the cross-loading itemized deletion process during the EFA analysis. Deletion of the MBEA domain corresponds with many previous studies [43–45]. Deletion of both the MBEA and CR domains, resulting in a two-factor MLQ model in hospital nurse setting was also previously suggested by Bycio and confirmed in this study [42]. The combination of MBEP and LF constructs into a “Lasseiz Faire-passive” (LFP) domain obtained in our EFA analysis was also supported in prior nurse leadership research, supporting the generalizability of this construct across cultures [40, 46].

### Organizational culture scale factor structure

CFA analysis on the organizational culture scale yielded a 13-item 4-factor structure after model refinement, the proposed structure was shown to apply within the study context, except for individual items referring to hospital policy management. As Hong Kong public hospitals are managed under an independent body, Hospital Authority (HA), most budgets, strategic planning, work manuals and human resource policies are set by HA [47]. Therefore, items such as “hospital adjusts budget scheme and process of work according to changing circumstances”, “hospital has detailed manual of work” and “hospital adopts top-down decision-making system” did not readily apply to our surveyed context were eliminated.

### Limitations

Despite inviting all full-time nurses in H1 to participate, the statistical power of the sample was inadequate. Thus, validation results were confirmed with data from H2. However, data from H2 was collected 6 months after H1 when external stressors (e.g. utilization rate fluctuations due to flu surge) may have differed. Nonetheless, factor structure and underlying constructs appear to be stable across time and hospital. Additionally, our data set comprised of self-reported leadership style ratings may be subject to social desirability bias. Thus, strict precautionary measures to protect confidentiality and anonymity were applied, such as emphasizing in information sheets and promotional seminar sessions at hospitals that completed surveys would be sealed in envelopes, stripped of personal identification and only collected, handled and opened by university research assistants not affiliated with the hospitals. The final report generated for hospitals will show only aggregate-level data.

## Conclusion

The N-LOC questionnaire was validated amongst nurses working in two distinctive Asian hospitals, yielding a 14-item 2-factor leadership and 13-item 4-factor organizational culture construct with satisfactory reliability. CFA results indicated that the proposed CVF organizational culture 4-factor structure was applicable to our sample but not the MLQ leadership 3-factor/9-factor structure. EFA revealed a 2-factor leadership style construct for our sample, which was named Confucius transformational and Laissez-Faire passive leadership. Transformational leadership traits already embedded in Confucius cultural values (self-sacrifice, stresses collective mission, instills pride) did not apply, where the new Confucius transformational construct which resembles LMX theory paternalistic leadership style is deemed more suitable in an Asian context. Study results demonstrated the importance of scale validation in cross-cultural adaptation, as underlying scale constructs may change with specific cultural and contextual factors. Future studies are encouraged to test the adaptation of this scale in other cultural and occupational settings.

## Additional file

Additional file 1: **Table S1.** Domains and descriptions of original 9-factor MLQ full range leadership theory and 4-factor CVF organizational culture questionnaire. (DOCX 17 kb)

## Data Availability

The datasets used and/or analyzed during the current study are available from the corresponding author on reasonable request.

## Abbreviations

A&E: Accident and emergency
APN: Advanced practitioner nurses
CFA: Confirmatory factor analysis
CFI: Comparative fit index
CR: Contingent reward
CVF: Competing Values Framework
DOM: Department operations manager
EFA: Exploratory factor analysis
EN: Enrolled nurses
GFI: Goodness-of-fit index
H1: Hospital 1
H2: Hospital 2
HA: Hospital Authority
IC: Individualized consideration
ICC: Intraclass correlation coefficient
ICC: Intra-class correlation coefficient
ICU: Intensive care unit
IIa: Idealized influence attributed
IIb: Idealized influence behavior
IM: Inspirational motivation
IS: Intellectual stimulation
LMX: Leader-Member Exchange theory
MBEA: Management by exception active
MBEP: Management by exception passive
MLQ: Multifactor Leadership Questionnaire
MLT: Multifactor Leadership Theory
N-LOC: Nurse Leadership and Organizational Culture Questionnaire
RMSEA: Root mean square error of approximation
RN: Registered nurses
RR: Response rate
SRMR: Standardized root-mean-square residual
TLI: Tucker-Lewis index
UIN: Unique identification code
UM: Unit manager
WM: Ward manager
