# Supplementary material for: Validation of the nurse leadership and organizational culture (N-LOC) questionnaire
Source: BMC Health Serv Res. 2019 Jul 9;19:469. doi: 10.1186/s12913-019-4290-z (PMC6617687; doi:10.1186/s12913-019-4290-z)
Supplement: Supplementary file 1 — Table S1. Domains and descriptions of original 9-factor MLQ full range leadership theory and 4-factor CVF organizational culture questionnaire. (DOCX 17 kb) [file 12913_2019_4290_MOESM1_ESM.docx]

**Table 1S.** Domains and descriptions of original 9-factor MLQ full range leadership theory and 4-factor CVF organizational culture questionnaire

| **Domain** | **Subdomain** | **# items** | **Description** |
| --- | --- | --- | --- |
| **Leadership** |  |  |  |
| Lasseiz-Faire | - | 4 | Does not lead, take accountability, use authority or make decisions unless major problems occur |
| Transactional | Contingent reward (CR) | 4 | Rewards employees when contractual obligations and tasks are fulfilled and completed |
|  | Management by Exception active (MBEA) | 4 | Leaders actively monitor employee mistakes and deviances, requesting employees to carry out corrections |
|  | Management by Exception passive (MBEP) | 4 | Leaders passively wait for mistakes and deviances to occur, then request employees to carry out corrections |
| Transformational | Individualized Consideration (IC) | 4 | Coaches employees to achieve through self-development and growth |
|  | Idealized Influence attributed (IIa) | 4 | Exhibits as role model and emphasizes on high ethical and moral standards. They are admired, respected and trusted by employees. |
|  | Idealized Influence behavior (IIb) | 4 | To spur employees in achieving goals through exhibiting extra effort |
|  | Inspirational Motivation (IM) | 4 | Creates shared vision and goals to arouse enthusiastic and motivated team spirit |
|  | Intellectual Stimulation (IS) | 4 | Encourages employees to tackle persistent old problems through innovation and creativity |
| **Organizational Culture** | |  |  |
| Group | - | 5 | People-centric and friendly culture emphasizes on teamwork, loyalty and participation |
| Rational | - | 5 | Results-centric and competitive culture that emphasizes on gaining market share and influence through demanding means |
| Developmental | - | 5 | Innovative and flexible culture that encourages changes, risk taking and experimentation |
| Hierarchical | - | 5 | Rigid and formal culture where efficiency is achieved through following procedural policies |
